# Supplementary material for: Machine Learning Accelerates Crystallization for Structure Determination
Source: Angew Chem Int Ed Engl. 2026 May 4;65(25):e1218503. doi: 10.1002/anie.1218503 (PMC13266958; doi:10.1002/anie.1218503)

checkCIF/PLATON report

Structure factors have been supplied for datablock(s) lcz066

THIS REPORT IS FOR GUIDANCE ONLY. IF USED AS PART OF A REVIEW PROCEDURE FOR PUBLICATION, IT SHOULD NOT REPLACE THE EXPERTISE OF AN EXPERIENCED CRYSTALLOGRAPHIC REFEREE.

No syntax errors found.   CIF dictionary   Interpreting this report

Datablock: lcz066

Bond precision: C-C = 0.0064 A   Wavelength=1.54184

Cell:	a=7.8834(1)	b=17.4573(3)	c=23.7253(3)
	alpha=90	beta=91.979(1)	gamma=90
Temperature:	100 K		

	Calculated	Reported
Volume	3263.20(8)	3263.20(8)
Space group	P 21/n	P 1 21/n 1
Hall group	-P 2yn	-P 2yn
Moiety formula	C23 H15 Ag3 F18 N6 O4	C15 H3 Ag3 F18 N6, C8 H12 O4
Sum formula	C23 H15 Ag3 F18 N6 O4	C23 H15 Ag3 F18 N6 O4
Mr	1105.02	1105.02
Dx,g cm-3	2.249	2.249
Z	4	4
Mu (mm-1)	15.677	15.677
F000	2120.0	2120.0
F000'	2130.49	
h,k,lmax	9,21,29	9,21,29
Nref	6668	6377
Tmin,Tmax	0.143,0.152	0.580,1.000
Tmin'	0.063	

Correction method= # Reported T Limits: Tmin=0.580 Tmax=1.000
AbsCorr = MULTI-SCAN

Data completeness= 0.956   Theta(max)= 74.500

R(reflections)= 0.0399(5751)   wR2(reflections)= 0.0915(6377)

S = 1.027   Npar= 517

The following ALERTS were generated. Each ALERT has the format
test-name_ALERT_alert-type_alert-level.
Click on the hyperlinks for more details of the test.

Alert level C

```
PLAT042_ALERT_1_C Calc. and Reported MoietyFormula Strings  Differ     Please Check
              Calc: C23 H15 Ag3 F18 N6 O4
              Rep.: C15 H3 Ag3 F18 N6, C8 H12 O4
PLAT215_ALERT_3_C Disordered F1        has ADP max/min Ratio .....        3.1 Note
PLAT215_ALERT_3_C Disordered F2        has ADP max/min Ratio .....        3.5 Note
PLAT215_ALERT_3_C Disordered F3        has ADP max/min Ratio .....        3.5 Note
PLAT215_ALERT_3_C Disordered F4        has ADP max/min Ratio .....        3.7 Note
PLAT215_ALERT_3_C Disordered F5        has ADP max/min Ratio .....        3.1 Note
PLAT215_ALERT_3_C Disordered F6        has ADP max/min Ratio .....        4.0 Note
PLAT431_ALERT_2_C Short Inter HL..A Contact  F00O     ..O00I     .      2.73 Ang.
                                               -1-x,1-y,1-z  =      3_466 Check
PLAT790_ALERT_4_C Centre of Gravity not Within Unit-Cell: Resd.  #          1 Note
              C23 H15 Ag3 F18 N6 O4
PLAT906_ALERT_3_C Large K Value in the Analysis of Variance ......      5.010 Check
PLAT911_ALERT_3_C Missing FCF Refl Between Thmin & STh/L=    0.600         58 Report
                9  1  0,   9  6  0,  -9  0  1,   9  0  1,   9  1  1,   9  2  1,
               -9  3  1,  -9  6  1,  -8 11  1,  -8  0  2,  -9  1  2,  -9  4  2,
               -9  5  2,  -9  6  2,  -8 11  2,  -9  0  3,  -9  1  3,  -9  2  3,
               -9  3  3,  -9  4  3,  -9  6  3,  -1 10  3,   4 17  3,  -9  1  4,
               -9  2  4,  -8  2  4,   8  2  4,  -9  0  5,  -9  1  5,  -8  2  5,
                   (   28 More Missing: see the .ckf listing file)
PLAT977_ALERT_2_C Check Negative Difference Density on H00L      .      -0.41 eA-3
PLAT977_ALERT_2_C Check Negative Difference Density on H01F      .      -0.31 eA-3
```

Alert level G

```
PLAT003_ALERT_2_G Number of Uiso or U(i,j)  Restrained non-H-Atoms          7 Report
PLAT007_ALERT_5_G Number of Unrefined Donor-H Atoms .............          1 Report
              H00L
PLAT083_ALERT_2_G SHELXL Second Parameter in WGHT  Unusually Large      13.52 Why ?
PLAT178_ALERT_4_G The CIF-Embedded .res File Contains SIMU Records          1 Report
PLAT186_ALERT_4_G The CIF-Embedded .res File Contains ISOR Records          3 Report
PLAT242_ALERT_2_G Low    'MainMol' Ueq as Compared to Neighbors of       C013 Check
PLAT242_ALERT_2_G Low    'MainMol' Ueq as Compared to Neighbors of       C014 Check
PLAT242_ALERT_2_G Low    'MainMol' Ueq as Compared to Neighbors of       C017 Check
PLAT242_ALERT_2_G Low    'MainMol' Ueq as Compared to Neighbors of       C01B Check
PLAT242_ALERT_2_G Low    'MainMol' Ueq as Compared to Neighbors of       C01D Check
PLAT242_ALERT_2_G Low    'MainMol' Ueq as Compared to Neighbors of       C01G Check
PLAT299_ALERT_4_G Atom Site Occupancy Constrained at ............        0.5 Check
              F1      F2      F3      F4      F5      F6
PLAT301_ALERT_3_G Main Residue  Disorder .............(Resd    1)         6% Note
PLAT720_ALERT_4_G Number of Unusual/Non-Standard Labels .........         65 Note
```

```
              Ag01    Ag02    Ag03    F004    F005    F006    F007    F008
              O009    F00A    F00B    F00C    F00D    O00E    F00F    F00G
              F00H    O00I    F00J    F00K    O00L    H00L    N00M    N00N
              F00O    N00P    N00Q    N00R    N00S    C00T    H00T    C00V
              C00W    H00W    C00X    C00Y    C00Z    C010    C011    H01A
              H01B    H01C    C012    C013    C014    C015    C016    H016
              C017    C018    C019    H01D    H01E    C01A    C01B    C01C
              H01F    H01G    H01H    C01D    C01E    H01I    H01J    H01K
              C01G
PLAT860_ALERT_3_G Number of Least-Squares Restraints ............         54 Note
PLAT883_ALERT_1_G Absent Datum for _atom_sites_solution_primary ..     Please Do !
PLAT912_ALERT_4_G Missing # of FCF Reflections Above STh/L=  0.600        232 Note
PLAT933_ALERT_2_G Number of HKL-OMIT Records in Embedded .res File         73 Note
               -9  0  1,  -9  0  3,  -9  0  5,  -9  0  7,  -9  0  9,  -9  0 11,
               -9  1  2,  -9  1  3,  -9  1  4,  -9  1  5,  -9  1  6,  -9  1  7,
               -9  1  8,  -9  1  9,  -9  1 10,  -9  1 11,  -9  1 12,  -9  2  3,
               -9  2  4,  -9  2  6,  -9  2  7,  -9  2  8,  -9  2 10,  -9  2 11,
               -9  3  1,  -9  3  3,  -9  3  5,  -9  3  7,  -9  3  8,  -9  3  9,
               -9  3 10,  -9  3 11,  -9  3 12,  -9  4  2,  -9  4  3,  -9  4  9,
               -9  4 10,  -9  5  2,  -9  5  7,  -9  6  1,  -9  6  2,  -9  6  3,
               -9  6  9,  -9  7  3,  -9  7  8,  -9  8  2,  -9  8  3,  -9  8  5,
               -8  0  2,  -8  2  4,
PLAT941_ALERT_3_G Average HKL Measurement Multiplicity ..........        3.3 Low
PLAT969_ALERT_5_G The 'Henn et al.' R-Factor-gap value ..........      3.131 Note
              Predicted wR2: Based on SigI**2  2.92 or SHELX Weight  8.90
PLAT978_ALERT_2_G Number C-C Bonds with Positive Residual Density.          0 Info
```

```
   0 ALERT level A = Most likely a serious problem - resolve or explain
   0 ALERT level B = A potentially serious problem, consider carefully
  13 ALERT level C = Check. Ensure it is not caused by an omission or oversight
  21 ALERT level G = General information/check it is not something unexpected

   2 ALERT type 1 CIF construction/syntax error, inconsistent or missing data
  13 ALERT type 2 Indicator that the structure model may be wrong or deficient
  11 ALERT type 3 Indicator that the structure quality may be low
   6 ALERT type 4 Improvement, methodology, query or suggestion
   2 ALERT type 5 Informative message, check
```

Validation response form

Please find below a validation response form (VRF) that can be filled in and pasted into your CIF.

```
# start Validation Reply Form
_vrf_PLAT042_lcz066
;
PROBLEM: Calc. and Reported MoietyFormula Strings  Differ     Please Check
RESPONSE: ...
;
_vrf_PLAT215_lcz066
;
PROBLEM: Disordered F1        has ADP max/min Ratio .....        3.1 Note
RESPONSE: ...
;
_vrf_PLAT431_lcz066
;
```

```
PROBLEM: Short Inter HL..A Contact  F00O     ..O00I     .       2.73 Ang.
RESPONSE: ...
;
_vrf_PLAT790_lcz066
;
PROBLEM: Centre of Gravity not Within Unit-Cell: Resd.  #          1 Note
RESPONSE: ...
;
_vrf_PLAT906_lcz066
;
PROBLEM: Large K Value in the Analysis of Variance ......      5.010 Check
RESPONSE: ...
;
_vrf_PLAT911_lcz066
;
PROBLEM: Missing FCF Refl Between Thmin & STh/L=    0.600         58 Report
RESPONSE: ...
;
_vrf_PLAT977_lcz066
;
PROBLEM: Check Negative Difference Density on H00L      .      -0.41 eA-3
RESPONSE: ...
;
# end Validation Reply Form
```

It is advisable to attempt to resolve as many as possible of the alerts in all categories. Often the minor alerts point to easily fixed oversights, errors and omissions in your CIF or refinement strategy, so attention to these fine details can be worthwhile. It is up to the individual to critically assess their own results and, if necessary, seek expert advice.

PLATON version of 17/09/2025; check.def file version of 17/09/2025

---

duplicate check

No duplication found

Datablock lcz066 - ellipsoid plot

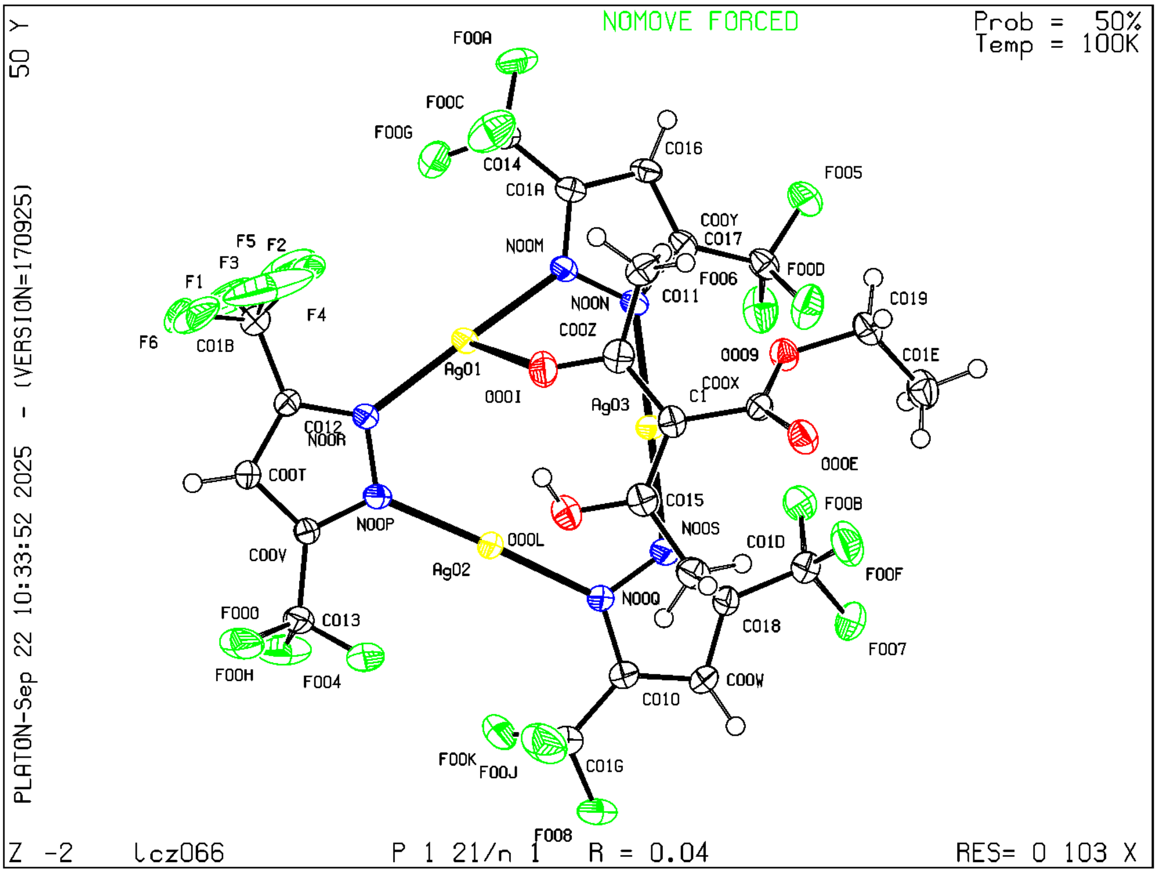

Supplement: Supplementary file 2 — Supporting File 2: anie72462‐sup‐0002‐DataFile.zip. [file ANIE-65-e1218503-s002.zip › supplementary_materials_crystallographic_data submit.zip!/cif_and_checkcif(1-114)/Ag3Pz3_66/Ag3Pz3_66_checkcif.pdf]
